# The Smartphone-Assisted Intervention Improved Perception of Nutritional Status among Middle School Students

**DOI:** 10.3390/ijerph17165932

**Published:** 2020-08-15

**Authors:** Yan-Hui Shen, Zheng Liu, Wen-Hao Li, Shuang Zhou, Jin-Hui Xu, Chu Jiang, Hai-Jun Wang

**Affiliations:** 1 Department of School Health, Beijing Haidian District Center for Diseases Prevention and Control, Beijing 100095, China; 18310307841@163.com (Y.-H.S.); Jack680623@126.com (C.J.); 2 Department of Maternal and Child Health, School of Public Health, Peking University, Beijing 100191, China; liuzheng@bjmu.edu.cn (Z.L.); 1811210197@bjmu.edu.cn (W.-H.L.); zhoushuang0601@bjmu.edu.cn (S.Z.); 1410306123@pku.edu.cn (J.-H.X.)

**Keywords:** student, perception, nutritional status, intervention

## Abstract

Misperception of nutritional status is common and hinders the progress of childhood obesity prevention. This study aimed to examine the effectiveness of a smartphone-assisted intervention to improve student and parental perception of students’ nutritional status (underweight, normal weight, overweight, obese). We conducted a parallel-group controlled trial with a non-randomized design in three junior middle schools of Beijing, China in 2019. One school was allocated to the intervention group and two schools to the control group. A total of 573 students (aged 13.1 ± 0.4 years) participated in the trial. The 3-month intervention included three components: health education sessions for students and parents, regular monitoring of students’ weight, and the provision of feedback via a smartphone application. Schools in the control group continued their usual practice. Primary outcomes included the student and parental accurate perception of students’ nutritional status. The percentage of students’ accurate perception of their own nutritional status in the intervention group increased from 49.0% to 59.2% from baseline to three months, whereas it decreased from 64.1% to 58.1% in the control group; the adjusted odds ratio (OR) between the two groups was 1.71 (95% confidence interval (CI): 1.13, 2.59). The intervention did not significantly improve parental perception of students’ nutritional status (*p* > 0.05). The study findings provided a brief approach for improving perception of nutritional status among middle school students.

## 1. Introduction

Childhood obesity is a global health concern. Childhood obesity can have both immediate and long-term adverse effects that can affect a child’s physical and mental health [1], educational attainment [2], and quality of life [3], and can predict obesity [4] and cardiometabolic diseases in adulthood [5]. From an economic perspective, investing in childhood obesity prevention is cost-effective if the benefits of lifetime health and the improvement of quality of life are taken into consideration [6]. However, progress in tackling childhood obesity has been slow and inconsistent [7,8,9], and the World Health Organization (WHO) has called for broader action to combat childhood obesity since 2017 [10]. 

Among multiple factors of childhood obesity, an accurate perception of nutritional status might play a crucial role in obesity prevention. For example, if students and their parents underestimate students’ nutritional status and inaccurately perceive students who are overweight to have a normal weight, students and parents may have less motivation to make lifestyle changes for the purpose of weight management [11,12].

Inaccurate perception of children’s nutritional status was common on the basis of findings from two recent systematic reviews, which revealed that 50.7% (95% confidence interval (CI): 31.1%~70.2%) of parents underestimated the nutritional status of children who were overweight or obese, and 14.3% (95% CI: 11.7%~17.4%) of parents underestimated their own child’s normal weight [13,14]. However, few interventions have been specifically designed and tested to improve the accuracy of parental perception of their children’s nutritional status [15,16,17], and to date, no interventions have been directly focused on improving students’ perception of their own nutritional status.

Weight monitoring, identified as an essential behaviour-changing technique for the success of weight management, has been utilized by several trials in the adult population [18,19,20,21]. However, little is known whether weight monitoring could be generalized to children and adolescents for improving perception of nutritional status and preventing obesity [22]. 

To bridge the gaps, this study aimed to examine the effectiveness of a smartphone-assisted intervention, focusing on monitoring and feedback techniques, to improve the perception of nutritional status. We hypothesized that the intervention would be effective to improve both student and parental perception of students’ nutritional status, which might be helpful in the effort to prevent obesity. 

## 2. Methods

### 2.1. Study Design

The study was a parallel-group controlled trial with a non-randomized design. The study was conducted in Haidian District in the northwest of Beijing from March to June in 2019. The study protocol was approved by the Ethics Committee of Haidian District Center for Disease Prevention and Control (201901) and was registered in Chinese Clinical Trial Registry (ChiCTR2000033348). 

### 2.2. Participants

Schools were eligible if they met the following criteria: (1) having not implemented obesity prevention programs in the past year, (2) the school principal was interested in the program and agreed to comply with the protocol, (3) a prevalence of obesity (data from regular monitoring of students’ physical health in Beijing) similar across the schools that were selected, and (4) not boarding or special schools (e.g., schools for students with talents or minority schools). Three schools were enrolled into the study. Students with eligible health conditions were included if written informed consent was obtained from both students and parents. 

### 2.3. Intervention

The intervention mainly included the three elements as described below.

### 2.4. Health Education

Research staff provided three health education sessions (each lasting for 30~40 min) for students and parents, respectively, during the first month of the intervention. Key messages of the health education included the health consequences of obesity, weight misperception and its association with lifestyle behaviors, and how to achieve a healthy body weight.

### 2.5. Regular Monitoring of Students’ Weight

Research staff monitored students’ height and weight in school approximately one month post-baseline (half way through the intervention). Based on this assessment as well as that at baseline, research staff calculated the change of body mass index (BMI; BMI = weight (kg)/(height (m))^2^) between the two time points for each student, selected out students at risk of excess weight gain (for students who were overweight or obese, an increase in BMI was considered to be excess weight gain; for students who were normal-weight, a change of nutritional status to overweight was considered to be excess weight gain), and then provided feedback to students and parents via telephone. To facilitate self-monitoring, students’ body weight was encouraged to be measured weekly by themselves.

### 2.6. Providing Feedback via a Smartphone Application

Research staff instructed parents to install the smartphone application (“Measure Your Nutritional Status”). Students and parents were asked to input students’ age, sex, weight, and height into the application immediately following baseline assessments, one-month after the baseline assessments, and each week during the monitoring. Then students and parents obtained automatic feedback from the application, including students’ current nutritional status (underweight, normal weight, overweight, or obese), and the distance to normal weight specific to students’ age, sex, and height if students’ nutritional status was not within the normal weight range. 

### 2.7. Control

Students in the control group continued their usual curriculum in school and did not receive any intervention sessions focused on obesity prevention.

### 2.8. Outcomes

Outcome measurements were conducted at baseline and at 3 months. The trained staff conducted baseline and follow-up measurements by using identical protocols and procedures. Table S1 summarizes the measurements taken during the study (including the instrument and method of assessment) and their associated outcome variables. 

Accuracy of student and parental perception of students’ nutritional status was examined by the cells that students and parents fell into within a 4 × 5 table (4 levels of actual nutritional status × 5 levels of perceived nutritional status) (Table S2). Actual nutritional status was classified based on students’ age- and sex-specific BMI [23], and included 4 categories: underweight; normal weight; overweight; and obesity. Perceived nutritional status included 5 levels as follows: very underweight; a little underweight; normal weight; a little overweight; and very overweight. Students and parents were classified as having an accurate or an inaccurate (underestimated or overestimated) perception of students’ nutritional status. 

The primary outcomes included the percentage of students’ accurate perception of their own nutritional status, and the percentage of parental accurate perception of their children’s nutritional status. The percentages of students’ underestimation or overestimation of their own nutritional status, and the percentages of parental underestimation or overestimation of their children’s nutritional status were assessed as secondary outcomes. The secondary outcomes also included students’ BMI, BMI Z-score [24], and the percentage of students in the contemplation (i.e., an individual who was not engaged in the behavior change but was thinking about becoming involved in the behavior in the near future), or action (i.e., an individual who has initiated some behavioral change) stage of a behavior change for the purpose of weight management.

### 2.9. Sample Size Estimation 

On the basis of our previous systematic review, we assumed that between-group difference in the percentage of students accurately perceiving their own nutritional status was 30%, the intra-cluster correlation coefficient was 0.02, and the attrition rate was 10% [9]. We estimated that 1 school and 217 students in the intervention group and 1 school and 217 students in the control group could provide 85% power with a = 0.05 to detect the assumed difference between the two groups. We actually recruited two schools in the control group as the number of students per school was not high enough, while our total recruitment of schools and students achieved the required sample size.

### 2.10. Statistical Analyses

The primary outcome analysis was conducted among students with a perception of nutritional status data available at both baseline and at 3 months, and students without the data were dropped as the missing rate was low (*n* = 30, 5.2%). Linear and logistic regression models were used to compare continuous and binary outcomes between the intervention and control groups, respectively. We used two models to adjust for potential confounders. For the primary outcome analysis, “Model 1” (Main Model) was used to adjust for the baseline value of the outcome, age, sex, whether students were overweight or obese (yes; no), whether parents accurately perceived their children’s nutritional status (for the outcome of students’ perception of their nutritional status) or whether students accurately perceived their own nutritional status (for the outcome of parental perception of their children’s nutritional status) (yes; no), and the primary caregiver of the students (mother; father; others); “Model 2” (Plus Model) was used to additionally adjust for maternal education level (high school or below; higher than high school), and whether the student was the only child in the family (yes; no) on the basis of “Model 1”. Multilevel models were not used because the number of clusters (schools) was too small (*n* = 3) for the models to be effective [25].

As sensitivity analyses, we tested whether the accuracy of parental perception of children’s nutritional status modified the intervention effect on the students’ accurate perception of their own nutritional status. We first included the interaction term (parental perception × group) into the regression model, and then conducted subgroup analyses to assess whether the intervention effect on the students’ accurate perception of their own nutritional status differed by the accuracy of parental perception of their children’s nutritional status. Similar interaction and subgroup analyses were also conducted to test the effect of sexual dimorphism on the primary outcomes. 

The level of statistical significance for the primary and secondary outcome analyses was two-sided at the 5% level of significance. Statistical analyses were performed using SPSS software, version 18.0 (IBM, Armonk, NY, USA).

## 3. Results

### 3.1. Baseline Characteristics

**Figure 1:**
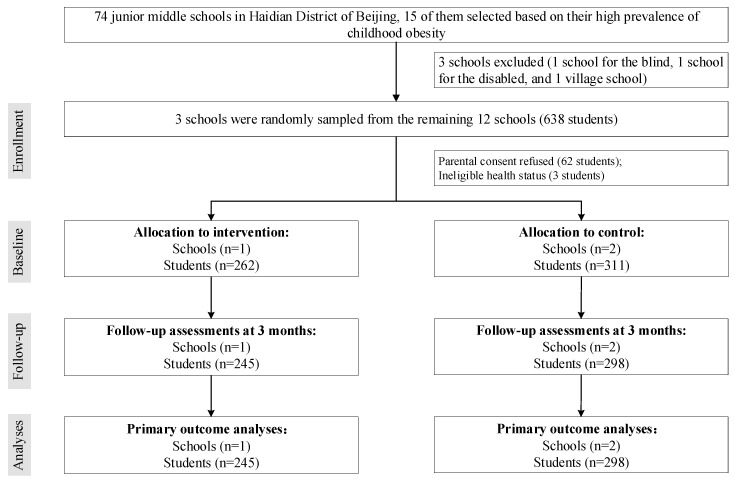
Trial profile.

Among the total of 638 students from 3 schools who were invited, 573 (89.8%) students were enrolled. Of these students, 543 (94.8%) completed the assessments at both baseline and 3 months and were included in the primary outcome analysis for students’ perception of their own nutritional status (Figure 1). 

Table 1 showed the baseline characteristics of the study population overall and by sex and study arms. Compared with boys, girls were more likely to be cared for by mothers (*p* = 0.043), and were less likely to be overweight or obese (*p* < 0.001). Students in the intervention and control groups did not differ statistically in age, weight, height, and BMI status (*p > 0.05*); however, the intervention group students were more likely to be the only child in the family, were less likely to be cared for by mothers or fathers, and their maternal education levels were higher (all *p* = 0.002).

### 3.2. Primary Outcomes

As shown in Table 2, the percentage of students’ accurate perception of their own nutritional status increased from 49.0% to 59.2% in the intervention group from baseline to 3 months, whereas it decreased from 64.1% to 58.1% in the control group. The odds ratio (OR) between the two groups in the percentage of students’ accurate perception of their own nutritional status was 1.71 (95% CI: 1.13, 2.59) in the “Main Model” and 1.75 (95% CI: 1.13, 2.69) in the “Plus Model”. Compared with the control group, the percentage of parental accurate perception of children’s nutritional status in the intervention group did not significantly increase at 3 months (*p* > 0.05). 

### 3.3. Secondary Outcomes

As shown in Table 2, the percentage of students who underestimated their own nutritional status decreased in the intervention group compared with the control group at 3 months (OR in the “Main Model”: 0.57; 95% CI: 0.34, 0.94). More students in the intervention group intended to initiate behavior change related to weight management (in the contemplation stage) in comparison with those in the control group (OR in the “Main Model”: 1.70; 95% CI: 1.04, 2.77), but the percentage of students actually initiating behavior change related to weight management (in the action stage) did not improve (*p* > 0.05). The students’ BMI and BMI Z-score did not significantly differ between the two groups (*p* > 0.05). Findings from the “Main Model” were broadly similar with those from the “Plus Model”.

### 3.4. Sensitivity Analyses

The intervention effect on students’ accurate perception of their own nutritional status was modified by the parental perception (*p* for interaction term = 0.004), and the effect was stronger in the subgroup of students whose parents inaccurately perceived their children’s nutritional status at baseline (OR: 2.61; 95% CI: 1.39, 4.91) compared to the other subgroup (1.07; 0.62, 1.85). There was a trend suggesting that students’ sex also modified the intervention effect on the students’ perception (*p* for interaction term = 0.086), with a stronger improvement in boys (2.33; 1.32, 4.10) than in girls (1.16; 0.63, 2.12). We did not observe a modification effect of students’ sex on the parental perception. 

## 4. Discussion

### 4.1. Main Findings

We observed that more than half (57.3%) of students did not perceive their nutritional status accurately at baseline. This smartphone-assisted intervention, focusing on the monitoring and feedback of students’ nutritional status, was effective in improving students’ weight perception. The intervention was also potentially beneficial to drive behavior change for weight management. More students in the intervention group intended to take action compared with those in the control group; however, we did not observe an effect of the intervention on increasing the percentage of students actually taking action for the purpose of weight management. The intervention did not improve parental perception and students’ BMI indices.

### 4.2. Comparison with Other Studies

To our knowledge, there have been few studies primarily exploring the effect of intervention on perception of nutritional status among children or parents to date [15,16,17]. A randomized controlled trial in the USA assessed the effect of an educational intervention and revealed that parental perception of their children’s nutritional status was not improved [15]. Future work is necessary to elucidate the key factors affecting the accuracy of parental perception. We have knowledge of two other studies reporting intervention protocols to improve parental perception of their children’s nutritional status, but intervention effects have not been reported [16,17]. 

Most previous studies of observational or intervention types were focused on parental perception of their children’s nutritional status [12,13,14,15]. Our study is novel in its additional focus on students’ perception of their own nutritional status. Our study population (i.e., the middle school students) also differed from that (i.e., preschoolers) of previous studies, as lifestyle behaviors of middle school students are independent from parents to some extent, while the behavior of preschoolers mostly relies on their parents. Therefore, the intervention effect on improving middle school students’ perception of nutritional status in this study might be important and could shed light on future similar studies for obesity prevention. 

### 4.3. Strengths and Limitations

This study has some limitations. Schools were allocated to the intervention or control groups based on practical considerations, rather than a randomization procedure, which might lead to a distribution imbalance of confounders between the two groups, and the observed effect size might thus have been biased. Additionally, the number of clusters (schools) in this study was small. However, findings of the study had been adjusted for multiple potential confounders, and the similar results derived from the “Main Model” with those from “Plus Model” further increased the reliability of our findings. 

Our study also has several strengths. First, the intervention is novel in its component of providing feedback of children’s nutritional status via a convenient tool of smartphone application, which is promising to overcome practical barriers and to reach wider populations [26]. Second, the attrition rate was only 5.2%, proving the feasibility of the intervention strategy to be translated into real-world settings. Third, the study measured an important mediator responsible for BMI change, i.e., the stage of behavior change for weight management, and the findings were likely to interpret the non-effective results of BMI indices. To enhance intervention effectiveness in reducing BMI indices, future interventions might borrow our experience in determining individuals’ stage of behavior change for weight management and implement stage-matched strategies for obesity prevention. For instance, for people in the action stage, strategies that address behavior-change skills may be highly effective, while for those in the pre-contemplation/contemplation stages, motivational strategies might be more important to encourage them to take action [27].

### 4.4. Public Health Implications

Our study has public health implications. First, the study found that the intervention effect on the students’ self-perception of nutritional status was stronger among those whose parents inaccurately perceived their children’s nutritional status at baseline, which suggests a prioritized target population for future intervention studies or public health practices. Second, boys and girls at the specific age of 13 years examined in this study probably differ in their awareness or attitudes towards nutritional status, and we observed that boys tended to benefit more from the intervention as to improving their perception of nutritional status. This highlights the need for a sex-specific public health approach in this area. Third, the intervention is simple to implement and lays a promising foundation for translation of the intervention approach into larger populations. Last but not the least, although monitoring and gaining feedback of students’ nutritional status as a stand-alone strategy in this intervention is not effective in reducing BMI indices, this component can work when it is incorporated into multifaceted programs for obesity prevention.

## 5. Conclusions

In conclusion, this smartphone-assisted and simple-to-implement intervention, which centered on monitoring and feedback, was effective in improving perception of weight status among middle school students. Future long-term and large-scale randomized controlled trials are needed to confirm the promising results of our study. Public health professionals could consider the brief approach used in this study to improve weight perception among middle school students. 

## Figures and Tables

**Table 1:** Baseline characteristics, overall and by sex and study arms ^※^.

Characteristics	All	Comparison between Sex			Comparison between Groups		
		Boys (*n* = 323)	Girls (*n* = 250)	*p* Value	Intervention (*n* = 262)	Control (*n* = 311)	*p* Value
Age, year	13.1 ± 0.4	13.1 ± 0.4	13.0 ± 0.3	0.275	13.0 ± 0.4	13.1 ± 0.4	0.275
Height, cm	161.8 ± 7.5	163.4 ± 8.2	159.6 ± 5.8	0.123	162.3 ± 7.6	161.3 ± 7.4	0.120
Weight, kg	55.3 ± 14.0	58.2 ± 15.5	52.0 ± 11.4	0.571	55.9 ± 14.4	55.2 ± 14.0	0.549
Primary caregivers, *n* (%)							
Mother	357 (62.3)	188 (58.2)	169 (67.6)	0.043	161 (61.5)	196 (63.0)	0.002
Father	151 (26.4)	91 (28.2)	60 (24.0)		59 (22.5)	92 (29.6)	
Others	65 (11.3)	44 (13.6)	21 (8.4)		42 (16.0)	23 (7.4)	
Education level of mothers, *n* (%) *							
High school or below	213 (40.0)	119 (40.3)	94 (39.5)	0.843	80 (32.9)	133 (45.9)	0.002
Higher than high school	320 (60.0)	176 (59.7)	144 (60.5)		163 (67.1)	157 (54.1)	
Nutritional status, *n* (%)							
Underweight	5 (0.9)	4 (1.2)	1 (0.4)	<0.001	3 (1.1)	2 (0.6)	0.878
Normal weight	368 (64.2)	174 (53.9)	194 (77.6)		165 (63.0)	203 (65.3)	
Overweight	101 (17.6)	76 (23.5)	25 (10.0)		48 (18.3)	53 (17.0)	
Obese	99 (17.3)	69 (21.4)	30 (12.0)		46 (17.6)	53 (17.0)	
The only child in the family, *n* (%) ^#^							
Yes	350 (63.2)	202 (65.2)	148 (60.7)	0.275	177 (70.0)	173 (57.5)	0.002
No	204 (36.8)	108 (34.8)	96 (39.3)		76 (30.0)	128 (42.5)	

^※^: Values were presented as mean ± SD for continuous variables or *n* (%) for categorical variables; *: Missing 40 values; ^#^: Missing 19 values.

**Table 2:** Intervention effect at 3 months.

	Intervention ^※^		Control ^※^		Model 1 (Main Model)		Model 2 (Plus Model)	
	Baseline	3 Months	Baseline	3 Months	Adjusted OR or Mean Difference (95% CI)	*p* Value	Adjusted OR or Mean Difference (95% CI)	*p* Value
Perception of nutritional status								
Students’ accurate perception of their own nutritional status *	120 (49.0)	145 (59.2)	191 (64.1)	173 (58.1)	1.71 (1.13, 2.59)	0.01	1.75 (1.13, 2.69)	0.01
Parental accurate perception of their children’s nutritional status ^#^	139 (59.1)	150 (63.8)	186 (64.4)	183 (63.3)	1.23 (0.82, 1.85)	0.33	1.12 (0.74, 1.71)	0.59
Students’ underestimation of their own nutritional status *	90 (36.7)	59 (24.1)	72 (24.2)	73 (24.5)	0.57 (0.34, 0.94)	0.03	0.56 (0.33, 0.94)	0.03
Students’ overestimation of their own nutritional status *	35 (14.3)	41 (16.7)	35 (11.7)	52 (17.4)	0.62 (0.34, 1.14)	0.12	0.61 (0.33, 1.15)	0.13
Parental underestimation of their children’s nutritional status ^#^	81 (34.5)	67 (28.5)	87 (30.1)	76 (26.3)	1.08 (0.68, 1.70)	0.74	1.17 (0.72, 1.89)	0.53
Parental overestimation of their children’s nutritional status ^#^	15 (6.4)	18 (9.0)	16 (5.5)	30 (12.2)	0.53 (0.27, 1.06)	0.07	0.50 (0.24, 1.04)	0.06
Stage of change ^#^								
The contemplation stage	77 (31.8)	90 (37.2)	110 (36.7)	99 (33.0)	1.70 (1.04, 2.77)	0.03	1.61 (0.97, 2.67)	0.07
The action stage	125 (53.0)	123 (52.1)	184 (63.0)	174 (59.6)	0.78 (0.52, 1.18)	0.24	0.89 (0.58, 1.38)	0.60
BMI indices								
BMI ^¶^, kg/m^2^	21.0 ± 4.3	20.7 ± 4.4	21.0 ± 4.3	20.7 ± 4.4	0.03 (−0.12, 0.18)	0.65	0.03 (−0.12, 0.18)	0.66
BMI Z-score ^Δ^	0.6 ± 1.3	0.3 ± 1.3	0.6 ± 1.2	0.4 ± 1.3	0.05 (−0.10, 0.20)	0.52	0.02 (−0.03, 0.07)	0.47

^※^ Values were presented as mean ± SD for continuous variables or *n* (%) for categorical variables. * *n* = 543; potential confounders in Model 1 included baseline outcome value, age, sex, whether students were overweight or obese (yes; no), whether parents accurately perceived their child’s nutritional status, primary caregiver of the students (mother; father; others), and in Model 2 additionally included maternal education level (high school or below; higher than high school) and whether the student was the only child in the family (yes; no). ^#^ *n* = 524, 542, 528 for the outcomes of parental perception, the contemplation stage, and the action stage; potential confounders in Model 1 included baseline outcome value, age, sex, whether students were overweight or obese (yes; no), whether students accurately perceived their own nutritional status, and in Model 2 additionally included primary caregiver of the students (mother; father; others), maternal education level (high school or below; higher than high school), and whether the student was the only child in the family (yes; no). ^¶^ *n* = 543; potential confounders in Model 1 included baseline outcome value, age, sex, primary caregiver of the students (mother; father; others), maternal education level (high school or below; higher than high school), and whether the student was the only child in the family (yes; no), and in Model 2 additionally included students’ perception of their own nutritional status (accurate; inaccurate), and whether students were in the action stage for behavior change (yes; no). ^Δ^ *n* = 543; potential confounders in Model 1 included baseline outcome value, primary caregiver of the students (mother; father; others), maternal education level (high school or below; higher than high school), and whether the student was the only child in the family (yes; no), and in Model 2 additionally included students’ perception of their own nutritional status (accurate; inaccurate), and whether students were in the action stage for behavior change (yes; no). Abbreviations: BMI = body mass index; OR = odds ratio.

## Supplementary Materials

The following are available online at https://www.mdpi.com/1660-4601/17/16/5932/s1, Table S1 Measurements and their associated outcome variables, Table S2 Correct, underestimated, or overestimated perception of student’s weight status on the basis of data from the student’s actual weight status and estimation of student’s weight status by students or parents.
