# Supplementary material for: The Smartphone-Assisted Intervention Improved Perception of Nutritional Status among Middle School Students
Source: Int J Environ Res Public Health. 2020 Aug 15;17(16):5932. doi: 10.3390/ijerph17165932 (PMC7459995; doi:10.3390/ijerph17165932)
Supplement: Supplementary file 1 [file ijerph-17-05932-s001.pdf]

Supplementary material List of supplementary tables

1. Table S1 Measurements and their associated outcome variables

2. Table S2 Correct, underestimated, or overestimated perception of student's weight status on the basis of data from the student actual weight status and estimation of student's weight status by students or parents

Supplementary Table 1 Measurements and their associated outcome variables

**Measurements**	**Instrument**	**Number of measurements at each time point**	**Method of assessment**	**Outcome variables**
Height	Stadiometer (Wujin RGT-140)	Twice	Measured to the nearest 0.1 cm	BMI and BMI Z-score$^{\Delta}$
Weight	Lever scale (Xiangshan EB839)	Twice	Measured to the nearest 0.1 kg	
Weight perception	One question asking students or parents to describe students' weight status as "*very thin*", "*a little thin*", "*normal weight*", "*a little overweight*", or "*very overweight*".	Once	Students finished the questionnaires in the classroom in the presence of the trained outcome assessors who can provide guidance and help.	Students' perception of their own weight status (*correct; incorrect*); parental perception of their children's weight status (*correct; incorrect*); Students' underestimation/overestimation of their own weight status (*yes; no*); parental underestimation/overestimation of their children's weight status (*yes; no*)

Stage of behavior change for weight management	The validated items measuring stages (in the action stage versus in the pre-action stage) of behaviour change for the purpose of weight management[#]	Percentage of students in the different stages (“preintenders”, “intenders”, “actors”) of behavior change for the purpose of weight management[*]

[Δ]Reference: de Onis M, Onyango A, Borghi E, Siyam A, Nishida C, Siekmann J. Development of a WHO growth reference for school-aged children and adolescents. Bull World Health Organ, 2007, 85: 660-667.

[#]Reference: Sarkin JA, Johnson SS, Prochaska JO, Prochaska JM. Applying the transtheoretical model to regular moderate exercise in an overweight population: validation of stages of change measure. Prevention Medicine 2001; 33: 462–9.

[*]"Preintenders": an individual who is not engaged in behaviour change and has no intention of becoming involved in that behaviour in the future; "Intenders": an individual who is not engaged in the behaviour change but is thinking about becoming involved in the behaviour in the near future; "Actors": an individual who has initiated some behaviour change.

Supplementary Table 2 Correct, underestimated, or overestimated perception of student's weight status on the basis of data from the student actual weight status and estimation of student's weight status by students or parents

Student actual weight status	Estimation of student's weight status (by students or parents)				
	Very underweight	A little underweight	Normal weight	A little overweight	Very overweight
Underweight	Correct	Correct	Overestimated	Overestimated	Overestimated
Normal weight	Underestimated	Underestimated	Correct	Overestimated	Overestimated
Overweight	Underestimated	Underestimated	Underestimated	Correct	Overestimated
Obese	Underestimated	Underestimated	Underestimated	Underestimated	Correct
